# The Telomerase Reverse Transcriptase Subunit from the Dimorphic Fungus *Ustilago maydis*

**DOI:** 10.1371/journal.pone.0109981

**Published:** 2014-10-09

**Authors:** Dolores Bautista-España, Estela Anastacio-Marcelino, Guillermo Horta-Valerdi, Antonio Celestino-Montes, Milorad Kojic, Erasmo Negrete-Abascal, Hortensia Reyes-Cervantes, Candelario Vázquez-Cruz, Plinio Guzmán, Patricia Sánchez-Alonso

**Affiliations:** 1 Centro de Investigaciones en Ciencias Microbiológicas, Instituto de Ciencias, Benemérita Universidad Autónoma de Puebla, Puebla, Puebla, Mexico; 2 Departamento de Ingeniería Genética, Centro de Investigación y de Estudios Avanzados, Irapuato, Guanajuato, Mexico; 3 Institute of Molecular Genetics and Genetic Engineering, University of Belgrade, Belgrade, Serbia; 4 Facultad de Estudios Superiores Iztacala, UNAM, Los Reyes Iztacala, Tlalnepantla, Estado de Mexico, Mexico; 5 Facultad de Ciencias Físico Matemáticas, Benemérita Universidad Autónoma de Puebla, Puebla, Puebla, Mexico; Tulane University Health Sciences Center, United States of America

## Abstract

In this study, we investigated the reverse transcriptase subunit of telomerase in the dimorphic fungus *Ustilago maydis*. This protein (Trt1) contains 1371 amino acids and all of the characteristic TERT motifs. Mutants created by disrupting *trt1* had senescent traits, such as delayed growth, low replicative potential, and reduced survival, that were reminiscent of the traits observed in *est2* budding yeast mutants. Telomerase activity was observed in wild-type fungus sporidia but not those of the disruption mutant. The introduction of a self-replicating plasmid expressing Trt1 into the mutant strain restored growth proficiency and replicative potential. Analyses of *trt1* crosses *in planta* suggested that Trt1 is necessary for teliospore formation in homozygous disrupted diploids and that telomerase is haploinsufficient in heterozygous diploids. Additionally, terminal restriction fragment analysis in the progeny hinted at alternative survival mechanisms similar to those of budding yeast.

## Introduction

Telomerase is a specialized ribonucleic protein complex that synthesizes the repetitive G-rich-DNA motifs constituting telomeres in most eukaryotic cells. The central enzyme components are the telomerase reverse transcriptase (TERT) protein subunit, which is a specialized reverse transcriptase, and the RNA template for telomere DNA synthesis (TR); both of these components are tightly regulated in normal cells [1], [2]. This enzyme plays an important role in telomere lengthening and has been intimately associated with cellular proliferation not only through its telomere-lengthening activity but also through its recently discovered non-telomeric roles [3], [4]. The reverse transcriptase subunit and RNA template are required for telomere synthesis, but *in vivo*, the TERT subunit is the limiting factor for restored telomerase activity in telomerase-downregulated cells. Therefore, most studies on telomerase regulation have focused on the TERT subunit gene [5], [6].

TERT subunit expression and activity have been detected in unicellular eukaryotes and metazoans. *TERT* can be regulated by diverse mechanisms, including both transcriptional regulation and posttranscriptional alternative mRNA splicing, multimerization, phosphorylation of the telomerase catalytic subunit and ncRNA interactions [7]–[9]. *TERT* gene expression and telomerase activity have been detected in immortal cells, such as cancer cell lineages and in germinal and pluripotent cells [10]–[12]; however, in most somatic cells, the *TERT* gene is gradually downregulated as cellular development progresses in the metazoan life cycle, and telomerase activity eventually becomes undetectable [11], [13]. Because conventional polymerases fail to replicate chromosome ends, telomerase downregulation results in telomere shortening in each replication round until a critical length is reached [14]. At this point, in telomerase-deficient cells, cellular proliferation ceases, senescence begins, and self-renewal capability decreases.

In *Saccharomyces cerevisiae*, the *EST2* gene encodes the reverse transcriptase subunit of telomerase, and its expression is tightly regulated throughout the yeast cell cycle. When *EST2* is mutated, yeast show a senescent phenotype, and their life span is reduced, as evidenced by an increase in cell death [15]. Eventually, survivors do appear through homologous recombination pathways, which cause rearrangements in telomeric and subtelomeric sequences that ensure the maintenance of telomere function [16]. Based on their telomere pattern, yeast survivors are classified into two types: Type I survivors, which exhibit tandem amplification of the Y' element followed by small tracts of telomeric C_1–3_A/TG_1–3_ DNA; and Type II survivors, which show very long and heterogeneous tracts of C_1–3_A/TG_1–3_ DNA at chromosome termini. The growth and maintenance of the two types of telomere structures have specific genetic requirements, involving more than a dozen genes [17]. In yeast cells, the programmed downregulation of *EST2* does not occur, and natural telomere attrition through successive rounds of DNA replication does not appear to be the main cause of replicative senescence. In this organism, replicative senescence has primarily been associated with the control of genetic pathways involving rDNA metabolism, mitochondrial dysfunction, and proteasome function [18]. The participation of telomere dysfunction by means other than programmed *EST2* downregulation in natural replicative senescence is currently under investigation. Other lower eukaryotes that undergo developmental transitions in their life cycle could be useful model systems to analyze the evolutionary pathways used to promote telomere maintenance and control by comparing their features with those of vertebrates or yeast-like organisms.

*Ustilago maydis*, a smut fungus that triggers the formation of tumors in maize, is a well-established model organism [19]–[22]. As a biotrophic pathogen, this fungus is an obligate parasite that depends on its host to complete its life cycle. Fungal infection is initiated with the recognition and mating of two compatible yeast-like cells, known as sporidia, on any of the aerial parts of the maize plant [23], [24]. Once plasmogamy occurs, the fungus enters into the plant tissue; tumor formation is then governed by a heterodimeric transcriptional regulator that triggers a dimorphic transition and pathogenic development and regulates mycelial growth and teliospore formation [25]–[27]. Regulatory cascades are initiated for a plethora of genes encoding transcriptional regulators, protein effectors, and virulence factors that subdue plants' defense responses; these processes result in tumor gall development, teliospore formation, and life cycle completion [28], [29]. In *U. maydis*, the TTAGGG motif composes the chromosomal end repeats [30]. Analysis of the terminal restriction fragment (TRF) by Southern blotting has indicated that *U. maydis* telomeres are maintained by telomerase [31]. In this study on telomere metabolism in this fungus, we report the identification and functional characterization of the *U. maydis* telomerase reverse transcriptase gene *trt1*.

## Results

### Identification and disruption of the Trt1 gene in *U. maydis*

**Figure 1:**
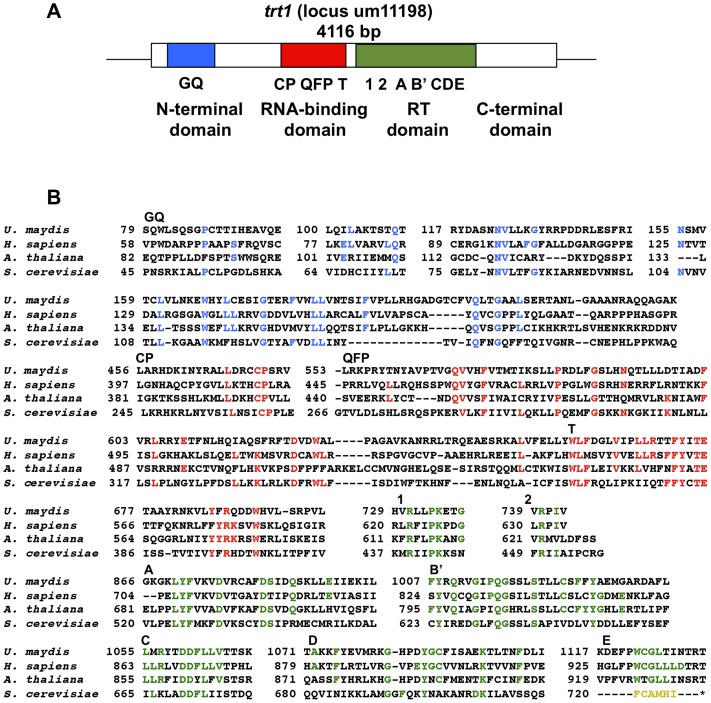
Structure of the *U. maydis* um11198 locus. The illustrative representation of the locus encoding the putative telomerase reverse transcriptase subunit (Trt1) of *U. maydis* is shown. (A) The open reading frame is depicted as a box, and the TERT domains are colored. The thin black lines represent the non-coding sequences located up- and downstream of the gene. (B) The conserved GQ (blue), RBD (red), and TR (green) domains of Trt1 are indicated above each highlighted alignment. The conserved residues are colored as in A. The sequences are from the representative organisms *Homo sapiens* (accession NP_937983.2), *Arabidopsis thaliana* (accession AF172097_1), and *Saccharomyces cerevisiae* (accession AAB64520.1), where the motif E does not align with other TERTs (asterisk). The aligned sequences used to define the motifs include at least 12 species, but only the representative organisms are shown.

Bioinformatics analysis of the annotated *U. maydis* genome (http://mips.helmholtz-muenchen.de/genre/proj/ustilago/) revealed that telomerase reverse transcriptase is encoded by a single, uninterrupted open reading frame (ORF). This ORF is predicted to yield a 1371-residue protein that contains all of the conserved domains found in the telomerase catalytic subunits of humans, plants, and fungi. These domains include the seven conserved RT motifs (1, 2, A, B′, C, D, and E) in the reverse transcriptase domain (shown in green, Fig. 1A and 1B) and the conserved motifs CP, QFP, and T from the RNA-binding domain, whose function is required for RNA subunit- binding (shown in red, Fig. 1A and 1B). In addition, the N-terminal region has the potential to house a GQ block (shown in blue, Fig. 1A and 1B).

**Figure 2:**
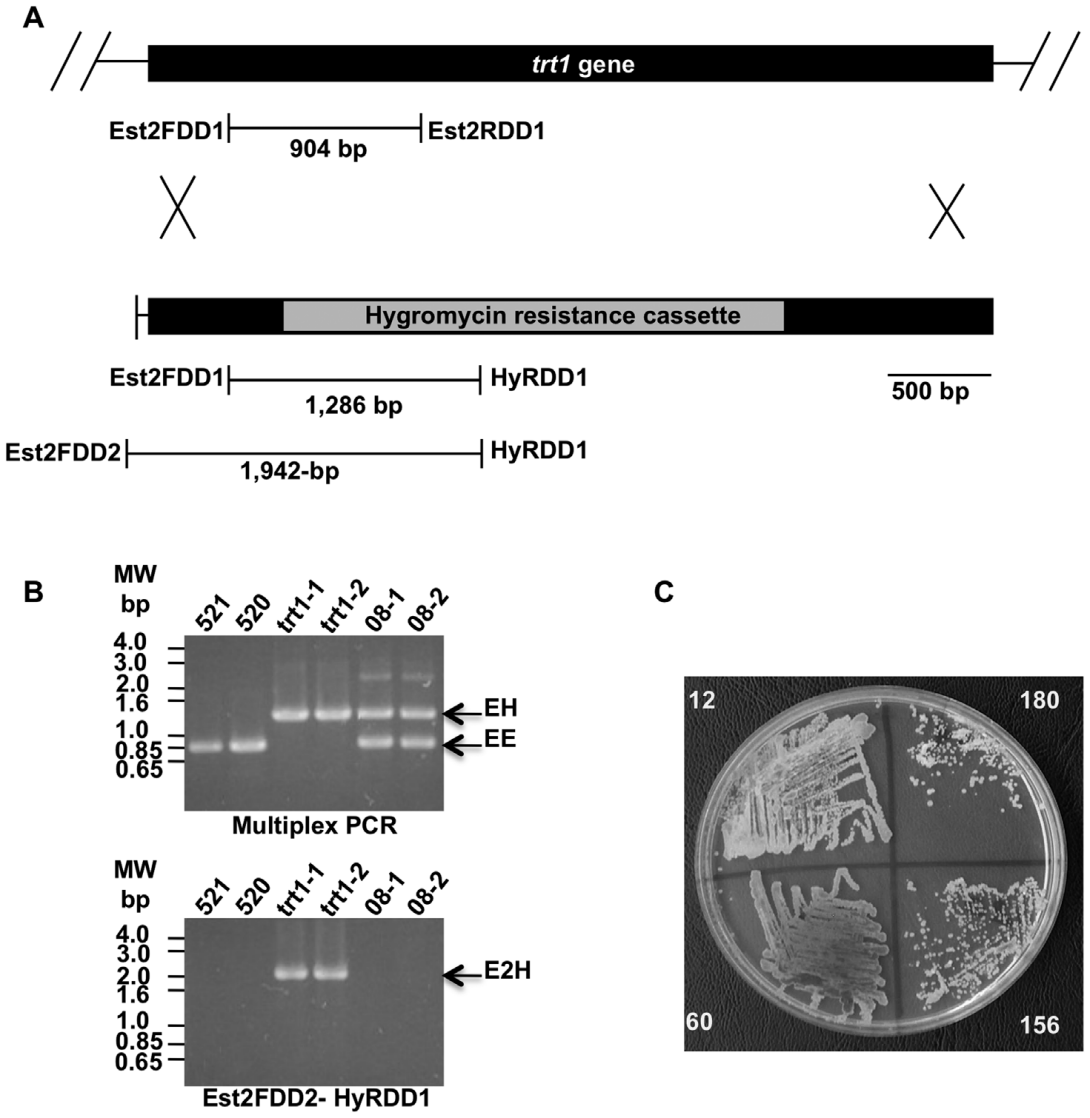
One-step disruption and recovery of *trt^-^* mutants. (A) The putative *trt1* gene is depicted as a black box (upper portion). The assembly containing the *hph* gene, which was used as selectable marker, is depicted as a light-gray box. The names of the primers used for the PCR assays and the lengths of the amplified products are indicated in the boxes below. The lines are to scale. (B) Multiplex-PCR amplification products of the *U. maydis* wild type strain 521, the 520 strain, trt1-1 and trt1-2 transformants after *trt1* disruption, and two clones of the 08 ectopic recombinant (upper gel). Disruption was verified via amplification of the fragment spanning part of the hygromycin-resistance gene and the *trt1* promoter (lower gel). EE indicates the DNA amplified using EST2 FDD1 and EST2 RDD1 (904 bp, wild-type pattern), and EH indicates the DNA amplified using EST2 FDD1 and HyR RDD1 (1,286-bp, *trt1*-disrupted or non-homologous recombinant pattern). E2H indicates DNA amplified with EST2FDD2 and HyR RDD1 (1,941-bp disrupted pattern). A molecular weight marker is shown on the left, and the strain name is shown in the top. (C) The phenotype of one disrupted strain, named *trt1-1*, was evaluated after 12, 60, 156, and 180 doubling periods. Small, dry, irregular, slightly hyper-pigmented colonies with low replicative potential were observed after 180 doubling periods. The number of doubling periods is shown on each quarter of the petri dish.

To confirm that *trt1* encodes the Trt1p subunit of *U. maydis* telomerase, we deleted this gene from the haploid 521 strain using standard one-step gene disruption methodology (Fig. 2A). Gene disruption was scrutinized by multiplex PCR using primers designed to detect the absence of the *trt1* coding region and its replacement with the hygromycin resistance gene, which was used as a selection marker (Fig. 2B, upper gel, lanes 3 and 4). Disruption was confirmed by the PCR amplification of a 1,942 bp fragment spanning the hygromycin resistance gene and regions outside the disruption cassette (Fig. 2B, lower gel, lanes 3 and 4). Ectopic recombination events were identified by a double-band electrophoretic pattern that corresponded to wild type and disruption cassette sequences (Fig. 2B, upper gel, lanes 5 and 6). The absence of *trt1* disruption was indicated by a lack of DNA bands in the DNA amplification pattern (Fig. 2B, lower gel, lanes 5 and 6). Subsequently, a representative mutant clone, trt1-1, was streaked after 12, 60, 156, and 180 generations in subculture passages (Fig. 2C). Poor growth and low replicative potential were observed, supporting the notion that replicative senescence occurs in the *U. maydis* trt1-1 mutant.

### Telomere length analysis in *trt1*-disrupted mutants

**Figure 3:**
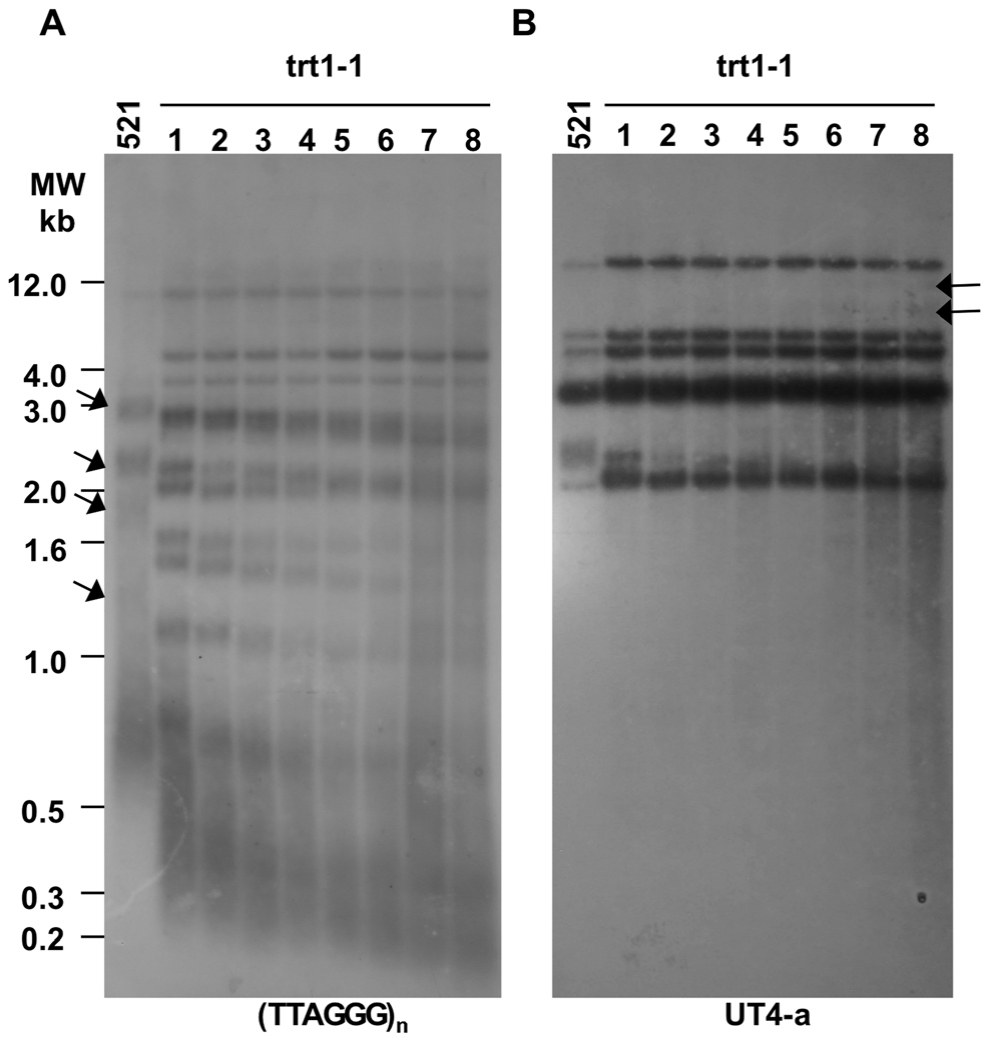
Terminal restriction fragment (TRF) analysis of a *trt1*-disrupted *U. maydis* mutant. (A) Samples of total DNA from the *U. maydis* 521 strain (150 ng) and trt1-1 mutant clone (500 ng) were digested with *Pst*I and quantified. The wild-type 521 strain was used as a control. A 36-generation series of samples was acquired from the trt1-1 mutant culture at the following population doubling times: 76 (lane 1), 112 (lane 2), 148 (lane 3), 184 (lane 4), 220 (lane 5), 256 (lane 6), 292 (lane 7), and 328 (lane 8). The DNA samples were hybridized to a TTAGGG probe as described in the Materials and Methods. (B) The nylon membrane was stripped and re-hybridized to UT4-a probe, an *Eco*RI/*Bam*HI fragment from the subtelomeric *UTASa* sequence, at high stringency conditions. The sizes (kb) are indicated on the left, and the arrows indicate the DNA bands showing changes in mobility or appearance.

To analyze the telomere length, trt1-1 mutant cell samples growing in continuous culture were acquired periodically (Fig. 3, lanes 1 to 8), and the DNA was isolated, quantified, and examined by Southern blotting. The 36-generation series initiated after approximately 76 doubling periods, when considerable telomere shortening had occurred compared with the wild-type strain (Fig. 3A, compare lanes labeled control ‘521’ and 1). Progressive telomere shortening continued as the replication rounds increased, and the hybridization signals began to fade at generation 256, when the sequences surveyed with the telomere probe were too short to yield a strong TRF signal (Fig. 3A, lanes 3 to 6). These results are consistent with previously reported telomere lengths [32]. The survivors, comprising a small group of telomerase-negative cells that employed homologous recombination-based pathways as an alternative method for telomere maintenance and proliferated past senescence, were obtained after 256 generations. Slight rearrangements in the hybridization pattern, such as changes in the size of signal bands (Fig. 3A, arrows on the left), their intensity, and the appearance of light smears along the last two lanes, were observed with the telomere probe at 292 and 328 generations (Fig. 3A, lanes 7 and 8).

To search for subtelomeric region rearrangements, we reprobed using the UT4-a probe from the subtelomeric *UTASa* gene [32]. Minor changes in the hybridization pattern that primarily correlated with a subtelomeric *UTASa*-like sequence shortening were observed (Fig. 3B, lanes 1 to 5); however, at later times in the survivor TRF pattern, two signal bands appeared at a high-molecular-weight resolution zone (Fig. 3B, lanes 7 and 8, upper arrows) as did smearing of the hybridization signals derived from subtelomeric sequences in survivor clones, which are detailed below.

### Telomerase activity in *U. maydis*

**Figure 4:**
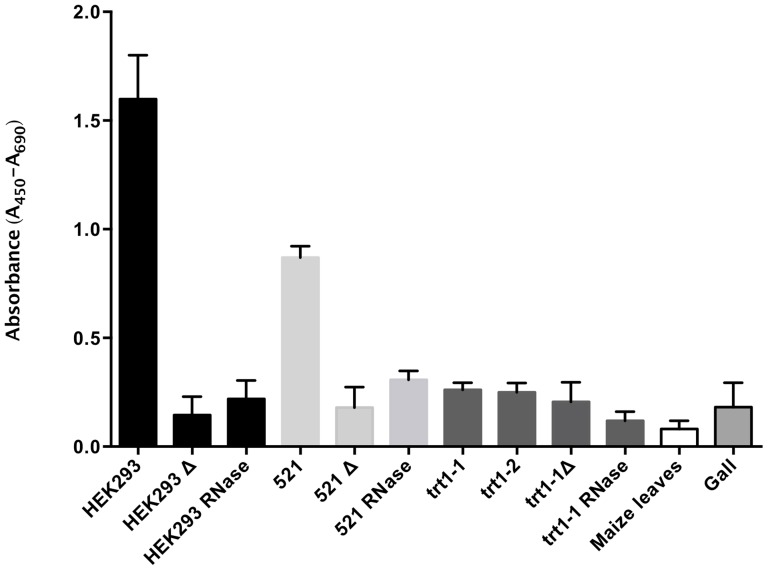
Telomere repeat amplification protocol (TRAP) analysis in *U. maydis*. Telomerase activity in wild-type and mutant strains was determined. The absorbance data were used to construct a graphical representation of the telomerase activity for the sporidia of *U. maydis* strains (either wild-type or *trt* ^-^). Tumor cells derived from the 521×520 cross and a plant control were included to evaluate and detect telomerase activity. The medians of the telomerase-positive control cells (HEK293) and the 521 wild-type strain were significantly different from the median of the treated negative controls (P<0.05); however, no significant differences were detected between the negative controls and the *trt1*-disrupted mutants. The samples heated to 85°C are indicated with Δ, and the RNase-treated samples are designated as RNase. Telomerase activity was also determined in tumors and maize leaves.

The telomerase activity in extracts from wild type sporidia was measured with TRAP-ELISA (telomeric repeat amplification protocol-enzyme-linked immunosorbent assay), a widely used assay developed to measure telomerase activity in mammalian cells. This assay is based on a one-step PCR procedure coupled with the chromogenic detection of telomeric repeat products. Extracts prepared from *U. maydis* 521 sporidial cells contained telomerase with a specific activity (activity per equivalent mass of protein) of approximately half the level found in control human HEK293 cells (Fig. 4, first and fourth bars; Table 1, first and second rows). Sample extracts from the HEK293 cell line and the *U. maydis* 521 strain heated at 85°C (Fig. 4, second and fifth bars) or treated with RNase (Fig. 4, third and sixth bars) had no activity compared with their corresponding non-treated positive controls (P<0.05). To determine whether telomere shortening in the trt1-1 clone correlated with a lack of telomerase activity, further TRAP-ELISAs were performed (Fig. 4, seventh, ninth and tenth bars; Table 1, third row). An additional independent *trt1* disrupted mutant (the trt1-2 clone) was also tested (Fig. 4, eighth bar; Table 1, fourth row). No significant differences (P>0.05) in activity were observed between the *trt1*-disrupted, heated, RNase-treated and gall cell extracts (Fig. 4, twelfth bar), indicating that these samples contained no detectable telomerase activity, as no activity was detected in the maize leaves (Fig. 4, eleventh bar; Table 1, fifth and sixth rows). Pairwise comparisons of the statistical means between the telomerase-positive and telomerase-negative samples using Mann-Whitney analysis revealed significant differences between the two groups of data (P<0.01). With this result accompanying the previous findings, we conclude that *U. maydis* synthesizes its telomeres with *trt1* telomerase activity.

**Table 1:** Determination of telomerase activity in *U. maydis* strains.

Cell type	Cell extract treatment*		
	None	Heated	RNase
HEK293	1.599	0.145	0.219
521 strain	0.870	0.180	0.307
trt1-1	0.261	0.206	0.118
trt1-2	0.249	N.D.	N.D.
Plant	0.081	N.D.	N.D.
Tumor (520×521)	0.182	N.D.	N.D.

The average absorbance values yielded by the telomerase activity in cell extracts are numerically expressed and shown in the chart. The absorbance for each sample was calculated according to the manufacturer's instructions and is shown. Heat- (85°C) and RNase-treated samples were used as negative controls. Telomerase activity was measured under the same conditions in the positive controls and tested samples. Telomerase activity was measured only in the mutant trt1-1; telomerase-negative samples (either from mutants, tumors, or plants) were not treated with heat or RNase, and their activity was not determined (N.D.). All of the experiments were performed at least three times.

* Media of at least three repetitions.

### The Trt1 activity is necessary for *U. maydis* teliospore formation

**Figure 5:**
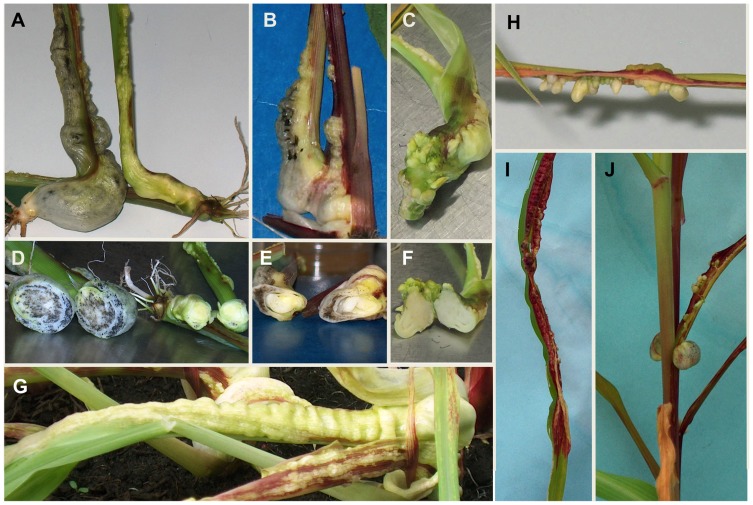
Analysis of the tumors caused by infection with mutant and wild-type *U. maydis* crosses. In (A and D), tumors caused by homozygous wild-type (left) and heterozygous *trt* ^−^/*trt* ^+^ (right) dikaryons on maize plants are shown at ripening day and after 17 dpi. Transversal sections depict the teliospore content. (B and E) Patched tumors were the most frequently observed trait in plants infected with heterozygous dikaryons, and scarce quantities of teliospores were observed. (C and F) Tumors derived from the homozygous *trt^−^* dikaryons were solid, teliospore-devoid tumors, which spontaneously healed when they developed in the leaves. (G and H) Tumor galls from heterozygous crosses at 26 and 30 dpi, respectively. (I and J) Galls growing on old leaves were present for the life of the leaf tissue or until the anthocyanin response of the plant eradicated the infection and the plant healed (I). However, new infections occasionally arose at the base of the primary infected tissues (J).

*In planta* crosses of wild type and telomerase-negative mutants were conducted to determine the effects of *trt1* disruption on the ability of *U. maydis* to complete its life cycle. Heterozygous or homozygous crosses for *trt^−^* were performed. In the heterozygous *trt^−^* crosses, the time when disease symptoms appeared in the infected plants was indistinguishable from the time of the wild-type control crosses (Fig. 5A and D; Table 2, ‘Symptoms’), but these crosses had lower teliospore production in the galls (Fig. 5B and E). In the heterozygous crosses, the number of plants with symptoms, immature tumors, or mature tumors was large (Table 2, see rows ‘Teliospore content in mature galls’, ‘Immature galls’ and ‘Mature galls’). Tumors were pale (Fig. 5G and H) or anthocyanin-rich (Fig. 5I and J). Homozygous crosses for trt1-1 (*a1b1*) were attained with trt1-53 (*a2b2*), a strain derived from the heterozygous cross trt1-1 ×520 (Table 3). In the homozygous *trt^−^* crosses, the disease symptoms were less frequent (Fig. 5C and F). Tumors arose from homozygous *trt1*-disrupted dikaryons, but the tumors appearing in the leaves healed (Table 2, ‘Tumor gall healing’). In galls that developed on the stalk, mature teliospores were absent (Fig. 5C and D right; Table 2, Teliospore content). Thus, we conclude that the *trt1* gene is required for teliospore formation.

**Table 2:** Time course of maize infection with mutant and wild-type strains of *U. maydis*.

	521×520^a^		trt1-1 X 520^b^		trt1-1 X trt1-53^c^	
Infection characteristics	Day	%	Day	%	Day	%
Symptoms appearing	5±2	57±8	5±2	88±12	5±2	25±1
Immature galls	11±1	57±7	12±2	72±12	12±8	19±6
Mature gall	15±2	60±6	21±2	76±7	20	none
Teliospore content in mature galls	15	+++/++++	26	+/+++ variable	25	none
Tumor gall healing	15	none	30	none	25	All of leaves
New tumor appearance	15	none	30	∼7	25	∼9

The wild-type 520 and 521 strains and their compatible derivatives trt1-1 and trt1-53 were used to perform *in planta* mating to determine the effects of the mutant *trt1* gene on cell cycle completion. The infectious process is listed on the left along with the disease signs and symptoms and lesion characteristics. The names of the compatible strains are shown above each column pair, and the mating genotype is displayed below. Column pairs were used for each compatible mating to show the percentages of plants assayed on the day of sign or symptom appearance.

a Wild-type X Wild-type.

b Mutant X Wild-type.

c Mutant X Mutant.

**Table 3:** *U. maydis* strains used.

Strain	Genotype	Source
521	*a1b1*	W. K. Holloman
trt1-1	*a1b1, trt1::hph*	This work
trt1-2	*a1b1, trt1::hph*	This work
520	*a2b2, nar1-1, met1-2*	W.K. Holloman
520trt1-1	a2b2, nar1-1, met1-2*	This work
trt1-53	*a2b2, trt1::hph*	This work, F1 from trt1-1 X 520
W204	*a1, b1, trt1::hph*	This work, F1 from trt1-1 X 520
T2	*a1, b1, trt1::hph*, pTrt1	This work, W204 transformed with pTrt1
521pNEB	*a1b1*, pNEBUC GE77+	This work

^*^Partially characterized.

### Insights into the telotype of the post-meiotic progeny

**Figure 6:**
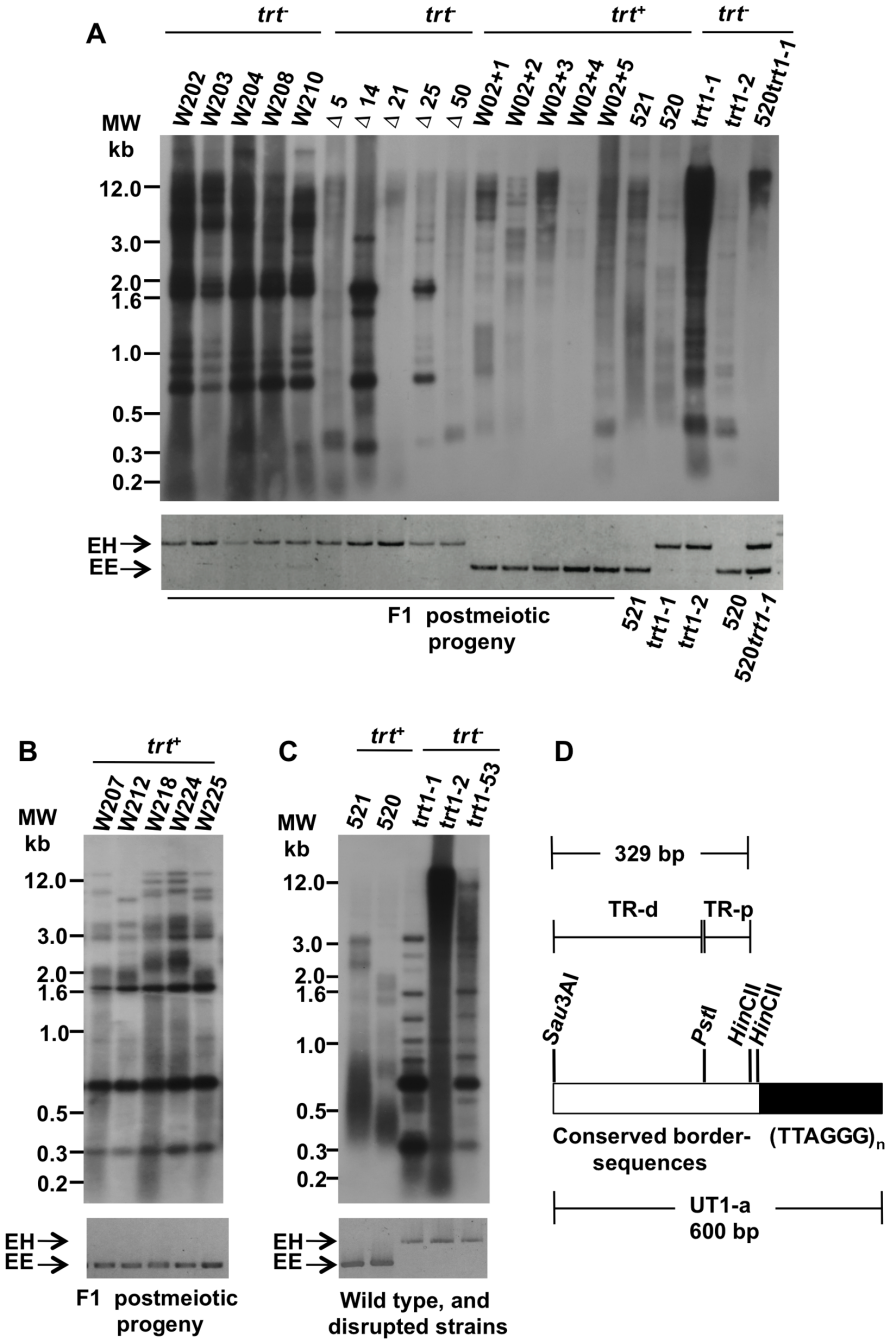
TRF length analysis from post-meiotic progeny. Hybridization patterns of the haploid progeny derived from *trt* ^−^/*trt* ^+^ diploids were evaluated to gain insight into their telotypes. (A) In the upper part of the autoradiogram, the 520× trt1-1 crosses resulted in W202, W203, W204, W208, and W210 *trt1*-disrupted progeny, whereas the 520trt1-1 heterozygous crosses resulted in W02+1 to W02+5 and the delta stains. (B) TRF pattern of W207, W212, W218, W224 and W225 *trt^+^* clones from the 520× trt1-1 cross, and (C) TRF pattern of parental strains, either wild-type or *trt1*-disrupted. The probe was UT1-a for (A) and (B) and (TTAGGG)_n_ for (C). The lower gel image shows the PCR amplification products used to identify the disrupted (EH) and wild-type (EE) *trt1* gene alleles in the different strains. A change in the sequence order of the analyzed samples is indicated at the right in (A). A molecular weight marker is shown on the left. (D) Diagram of the telomeric region used as UT1-a probe. The fragment is a *Sau*3A1 terminal restriction fragment is composed of 37 copies of the TTAGGG telomeric repeats (black box), and a *Hinc*ll- *Sau*3A1segment (open box) containing: TR-p, a 84 bp *Pst*I-*Hinc*ll fragment adjacent to the TR; and TR-d, a 245 bp *Sau*3Al-*Pst*l fragment of the TAS. Black lines above and under the boxes indicate the fragment lengths.

To investigate telomere length in the meiotic products of a heterozygous trt1-1 ×520 cross, we germinated the resulting teliospores on solid medium in the presence or absence of hygromycin. These progeny were designated as F1. Total DNA was extracted from selected F1 progeny, and PCR was used to search for the inherited *trt1* allele (Fig. 6A and B, lower panels). Five telomerase-negative strains derived from the heterozygous trt1-1 cross, referred to as W202, W203, W204, W208, and W2010, and five telomerase-positive strains from the same cross, named W207, W212, W218, W224 and W225, were selected for PCR analysis (Fig. 6A, lanes 1 to 5 and B, see legends in upper panels). We also performed heterozygous crosses of wild type and *trt1*-disrupted clones with the 520trt1-1 strain. This strain is an *a2b2*, telomerase-positive transformant clone with limited proliferative potential, affected telomere length maintenance and a TRF pattern similar to that of trt1-2, (Table 3; Fig. 6A, lane 20). Five telomerase-negative strains, designated here as delta (Δ), and five telomerase positive strains, referred to as W02+1 to W02+5, were selected and analyzed (Fig. 6, lanes 6 to 10 and lanes 11 to 15, respectively, see upper panel). The analyzed telomerase-negative strains produced a 1,286-bp amplified fragment that spanned parts of the *trt1* and *hph* genes, whereas the telomerase-positive strains produced a 904-bp amplified fragment from the *trt1* gene (EE; Fig. 6A to C, lower panels).

The terminal restriction fragment length was then analyzed in the F1 progeny mentioned above, the wild-type 521 and 520 strains, the disrupted derivatives trt1-1 and trt1-2, and the 520trt1-1 strain. This experiment used the UT1-a probe, a *Sau*3*A*I terminal restriction fragment that spans 240 bp from the telomeric repeated TTAGGG motif and 360 bp from a highly conserved sequence located adjacent to telomeric repeats and interspersed with telomere associated sequences (Fig. 6D) [30]. The latter element has also been previously reported to comprise TR-p + TR-d sequences, which together form a 329-bp *Sau*3AI-*Hin*cII DNA fragment riven by the *Pst*I recognition and excision site [27]. Two principal hybridization patterns were obtained from the progeny TRFs. One pattern exhibited abundant amplification of the telomeric sequences and its associated sequences, showing intense hybridization signals mainly at resolution zones around 0.3, 0.7, and 1.7 kb (Fig. 6A, lanes 1 to 5, Δ14, Δ25, and trt1-1; Fig. 6B, W207, W212, W218, W224 and W225). The other pattern was composed of a mixture of wild-type and mutant hybridization signals from the chromosomal terminal sequences, which were long, heterogeneous, and variable in intensity (Fig. 6A, Δ5, Δ21, Δ50, and W02+1 to W02+5, and 520trt1-1). The TRF patterns were similar between the *trt^+^* and *trt^-^* disrupted strain progenies of the trt1-1 ×521 cross (compare Fig. 6A, W202, W203, W204, W208, and W210 with; Fig. 6B, W207, W212, W218, W224 and W225), as were similar those of *trt^+^* and *trt^-^* progenies from heterozygous 520trt1-1 crosses (In Fig. 6A, compare W02+1 to W02+5 with Δ5, Δ21 and Δ50). The preservation of the hybridization pattern from the parental survivor across several generations in the telomerase-positive progeny from heterozygous crosses suggests the haploinsufficiency of the TERT component, which will be discussed later. In all strains, the PCR amplification patterns of the wild-type and *trt1*-disrupted alleles are shown at the bottom of Figure 6.

### Extrachromosomal complementation of *Trt1* mutants

**Figure 7:**
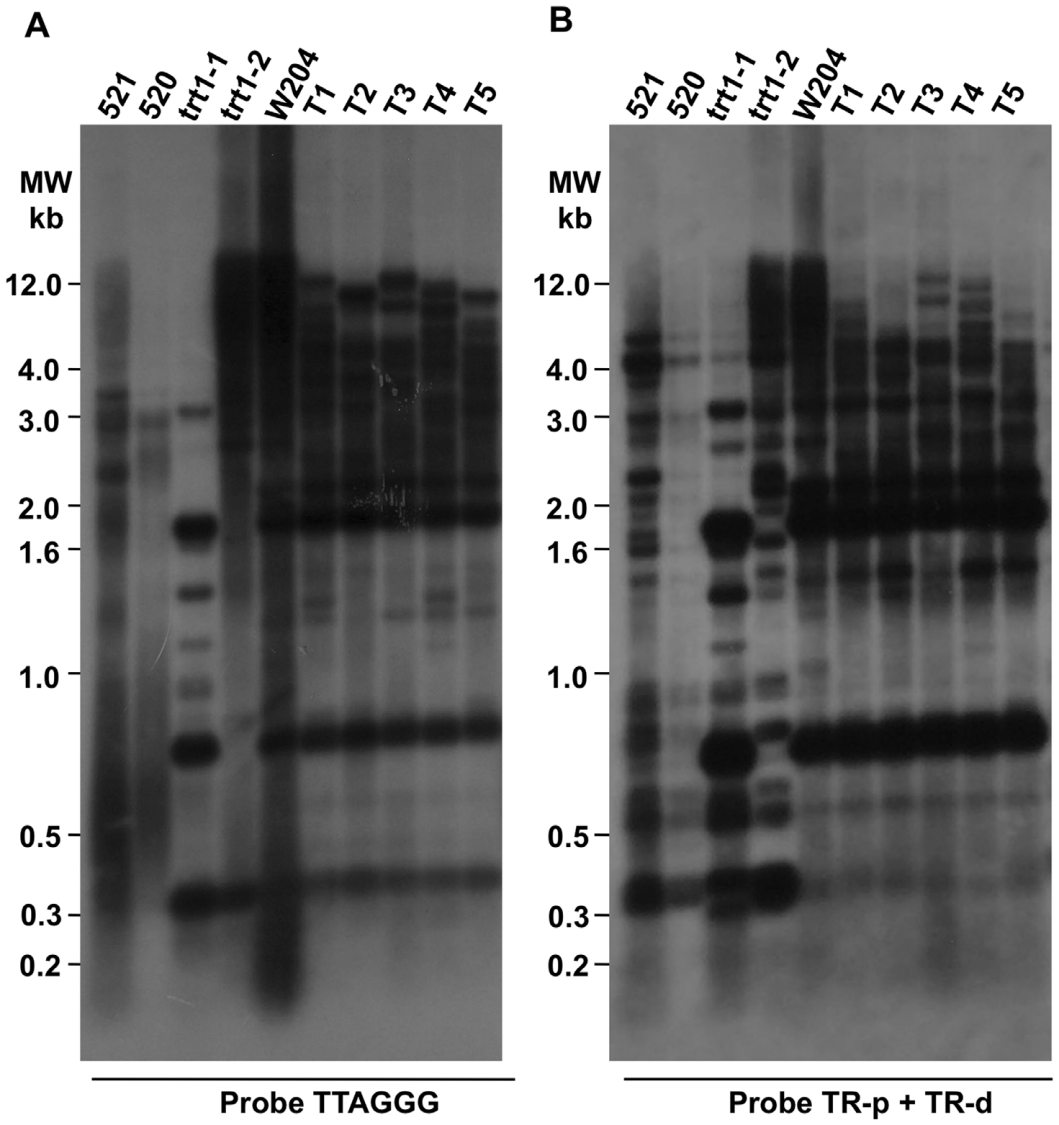
Analysis of the effects of *trt*+ restoration in pTrt1 *U. maydis* transformants. The telomere length distribution in the telomerase-deficient strain W204 was assessed by Southern blotting TRF after reintroduction (200 doubling periods) of *tert1* in the pTrt1 *U. maydis* transformants as described above. (A) The TRF hybridization pattern of parental 521 (lane 1), 520 (lane 2), *trt1*-disrupted mutants trt1-1 (lane 3) and trt1-2 (lane 4) strains, the progeny derivative W204 (lane 5), and five of its W204-derived clones (T1 to T5, lanes 6 to 10) were analyzed using telomere sequences (TTAGGG) that were ^32^P- labeled at 17 kBq/ml as probes. (C) The filter was stripped and re-hybridized to TR-p + TR-d sequences ^32^P- labeled as a probe. The strain names are shown above the autoradiography. A molecular weight marker is shown on the left.

The telomerase-negative survivor W204 strain, which was derived from the 520× trt1-1 cross, and the wild-type 521 strain were transformed with self-replicating plasmids expressing Trt1 (pTrt1) or a vector alone and were selected with carboxin resistance. The acquisition of pTrt1 into W204 transformants (T1 to T5) was confirmed with drop-plate assays (Fig. S1A) and with the genetic re-transformation of *E. coli* XL1-Blue MRF' with total DNA extracted from clones (data not shown). PCR amplification of DNA sequences from pTrt1 indicated that the plasmid was present in all five clones (Fig. S1B). Transcriptional expression of chimeric *trt1* was examined in three transformants (T1 to T3) by RT-PCR as described in Material and Methods section. The detection of *trt1* transcripts in the transformant clones suggested a functional complementation of the *trt1*-disrupted W204 mutant that depended on the exogenous chimeric gene (Fig. S1C). Regaining telomerase activity in the three transformant clones confirmed our assertion (Fig. S1D); however, as shown in the Table S1, telomerase activity varied between the T1 to T3 tested strains after complementation (Table S1, rows 2, 5, 6 and 7). Trt1 expression restored the replicative potential and growth rate to almost normal levels in the W204 derived strain T1 and decreased the growth rate in the 521 strain (Table 4). A slight decrease in intensity in the telomere signals from the genomic hybridization patterns of the pTrt1 transformants was observed after 200 doublings compared with the untransformed W204 strain (Fig. 7A, lanes 5 to 10). This change was accompanied by minor telomeric and subtelomeric rearrangements in the banding patterns (Fig. 7B, lanes 5 to 10), but no modifications were observed after 400 generations in 521 transformants with the pTrt1 or vector alone (data not shown). Thus, we assume that the deficient W204 strain was complemented with pTrt1.

**Table 4:** Extrachromosomal *trt1* complementation and growth rate restoration.

Strain	Transformant condition	Growth rates in min (hours)
521	Non-transformed	80 (1∶20 h)
	Transformed with pTrt1	285 (4∶45 h)
	Transformed with pNEBUC-GE77+	216 (3∶36 h)
W204	Non-transformed	815 (13∶35 h)
	Transformed with pTrt1 (T1)	122 (2∶02 h)
	Transformed with pNEBUC-GE77+	N.D.

After restoring the replicative potential by *trt1* reintroduction, the growth rate was analyzed in the transformants with the same method used for the non-transformed controls. The strains analyzed are shown on the left, and the plasmids used to transform these strains are indicated at the top. In parentheses, minutes were changed to hours.

### Time course of chromosome end degradation in *U. maydis*

As the sequences on or near the chromosome termini shorten in telomerase-deficient cells, a time course assay of *Bal*31 digestion was performed. This assay compared the pattern of telomere loss by telomerase deficiency to the pattern of telomere loss by TRF exonuclease treatment in the 521 as well as in the trt1-1 survivor, derivative W204 and complemented T2 clones. For the 521 strain, long smears corresponding to the (TTAGGG)_n_ hybridization pattern and extending from 0.3 to 3 kb were accompanied by six discrete bands in the resolution zone that ranged from 2 to 12 kb after UT4-a-probe hybridization in the autoradiogram at time 0 (Fig. 3B and S2A). The telomere smears gradually shortened (Fig. S2A, 30 min) or faded (Fig. S2A, 60 and 90 min), whereas the UT4-a hybridization signals remained insensitive to *Bal*31 exonuclease treatment during the 90-min treatment period (Fig S2A, 0 to 90 min), with exception of a 2.5-kb band. The requirement of a longer digestion time to erode the UT4-a bands reflects the inner localization of these sequences within the chromosomes. After membrane stripping and rehybridization to ribosomal rDNA sequences, the hybridization signals did not change on the blots because the internally located genes had no sensitivity to *Bal*31 (Fig. S2B, 0 and 90 min).

In the telomerase-negative survivor trt1-1, W204 and T2 transformant, strong hybridization signals for telomeric DNA were distributed from 0.3 to 12 kb (Fig. S2C, S2D and S2E, respectively). Hybridization bands of approximately 0.6 kb in the *trt1*-disrupted mutant trt1-1 (Fig. S2C, 0 min) and 4 kb from complemented T2 strain (Fig. S2E, 0 min) were partially sensitive to *Bal*31 action at short incubation times (Fig. S2C, 10 min, arrow and Fig. S2E, 20 and 30 min, arrow), whereas the remaining hybridization signal was insensitive to exonuclease digestion at longer incubation times (90 min, data not shown).

## Discussion

Here, we described the results of the disruption of the locus um11198 (MUMBD) in the *U. maydis* genome, which harbors the putative telomerase reverse transcriptase (*trt1*) gene. Alignment analysis indicated that it encodes a protein with all of the characteristic TERT domains (TEN, TRBD, RT, and CTE) in the same order and distances reported for other TERTs [33], [34].

After identifying telomerase activity in cell extracts of *U. maydis*, the *trt1* gene was disrupted to confirm the induction of telomere erosion by a lack of telomere maintenance; the replicative senescence and loss of telomere repeats found in the mutant trt1-1 clone agreed with the previously reported absence of telomerase activity [35]. The appearance of slight smears in the DNA hybridization pattern of trt1-1 with the UT4-a subtelomeric probe suggested the presence of chromosome termini alterations of the mutant after 292 replication rounds. Similar changes have been reported to occur in telomerase-negative yeasts through recombinational pathways [35]. The development of telomerase-negative survivors in the trt1-1 mutant was confirmed after several subsequent passages.

No telomerase activity was found in the trt1-1 and trt1-2 mutants compared with the 521 strain or a human positive control. The absence of telomerase activity, the presence of telomere shortening, and the senescent phenotype coupled with slow growth and poor replicative potential of the trt1-1 clone support our assertion that *trt1* is the reverse transcriptase catalytic subunit of the *U. maydis* telomerase.

In our TRF analysis, long heterogeneous tracts of telomeric sequences were observed in the trt1-2 mutant and in most progeny derived from heterozygous crosses of 520trt1-1 with 521 or the *trt1*-disrupted mutant. These TRF analyses resemble those characterized in the Type II survivor pattern of the *S. cerevisiae est2* mutants [36]. However, the excessively amplified tracts of telomere-associated sequences linked to short segments of telomeric sequence that occurred in trt1-1 and trt1-53 match the Type I survivor pattern [36]. Although it is tempting to propose that trt1-2 progeny are similar to Type II *S. cerevisiae est2* mutant survivors because of the excessive telomeric repeat sequence lengthening [35], it is more difficult to match trt1-1 and its progeny with the Type I survivor pattern because the length and identity of the amplified TAS by homologous recombination requires further characterization [35].

*U. maydis* and yeast survivors share similar characteristics: (a) the telomere structure and organization of mutant survivors prevail in the postmeiotic progeny from heterozygous diploids, as previously described [37], and (b) the inherited telomeric structure is preserved in haploid progeny for many generations, in *trt^+^* or *trt^-^*, regardless of the inheritance of the functional *trt1* allele in haploid cells, which occurs in yeast and mice [16], [38]. These similarities were unanticipated because of the distant relationship between these two fungal species.

Gall development from homozygous crosses of *trt^−^* mutants *in planta* suggests that the mechanisms for pathogenic development remained intact in these organisms. However, the lack of teliospore formation in these tumors indicates that Trt1 is indispensable for fungal life-cycle completion. This condition resembles the critical role of telomerase activity in the production and fertility of germinal cells in mammalian males and in the proliferation of human stem cells [10], [39], [40]. The delay in gall development may result from low mutant cell viability, the production of lower quantities of virulence factors, or a plant-cell/mutant growth ratio that influences the plant infection schedule, as the growth rate of the plant host is known to influence the pathogenic potential of *U. maydis* [41]. The roles of Trt1 in mycelium differentiation should be explored to explain the defective fungal differentiation process in the telomerase-negative mutants.

Insufficient telomerase activity was also found to affect telomere maintenance in the *trt^−^*/*trt^+^ U. maydis* heterozygotes. The lack of telomere pattern restoration observed in the telomerase-positive progeny after passaging through meiosis suggests Trt1 haploinsufficiency (Fig. 6B), which has been reported for humans, mice, and yeasts that are heterozygous for TERT or TR subunit null mutations [38], [42], [43]. Trt1 haploinsufficiency may also be responsible for the teliospore-lacking tumors produced by the *trt^−^*/*trt^+^* heterozygous crosses.

Introduction of the plasmid pTrt1 but not pNEBUC-GE77+ into the W204 strain yielded transformants, reinforcing our affirmation of an effective complementation of W204 with the exogenous copy of *trt1* in pTrt1. The decrease in intensity of the hybridization signal emitted by similar quantities of DNA from both the complemented strains and the telomerase-positive progeny suggested that a progressive reduction of TAS copies occurred after 200 doubling periods, resulting in a gradual reversion to the wild-type telomeric pattern. However, the replicative potential and growth rate were not fully recovered in the transformants. In contrast, a complete restoration of the growth rates and replicative potential occurs after the reintroduction of *EST2* to telomerase-null yeast mutants [44]. The lack of complete restoration observed in *U. maydis* may be a consequence of limitations in *trt1* expression.

Our analysis of the telomere structure also provided insight into the telomere maintenance that occurs at chromosome ends. A time course of *Bal*31 digestion of the telomeric DNA from the 521 strain demonstrated not only that (TTAGGG)_n_ and UT4-a sequences are located mainly on the chromosome ends but also that the 521 strain and trt1-1 share similar patterns of telomere attrition post-*Bal*31 digestion and after several passages in cell culture. The telomeric and subtelomeric DNA sequences of the W204 strain were more resistant to *Bal*31 digestion than those of its pTrt1 transformant derivative T2. The exogenous reintroduction of *trt1* seemed to attenuate DNA resistance to exonuclease action. Thus, we hypothesize that exogenous Trt1 contributes to the prevention of additional telomere enlargement via recombinational pathways; however, further studies should be conducted before comparing these findings with other models. Moreover, the survival mechanisms of the telomerase-negative *U. maydis* strains appear to rely on recombinational events, which also deserve further study and were recently proposed for telomerase-positive strains [45].

Finally, we assume that the *U. maydis* telomerase reverse transcriptase gene has been structurally and functionally characterized at the complementation level, but further analysis regarding telomerase function in this lower eukaryote remains to be performed.

## Materials and Methods

### Plasmids, strains, and mating assays

*E. coli* XL1-Blue MRF' (Stratagene, La Jolla, CA, USA) were used as the receptor strain for plasmid construction. Unless otherwise specified, this strain was grown overnight (O/N) at 37°C with aeration in LB medium supplemented with 100 µg/ml ampicillin when necessary. The plasmids used were as follows: pBluescript KS- (Stratagene), pTrcHis 2A (Invitrogen, Carlsbad, CA, USA), pCM1007, which contains a *trt1* disruption cassette and was kindly provided by Dr. W.K. Holloman (Weill Cornell Medical College, NY, USA); pUBLEX1 [46], which was supplied by Dr. Fidel Guevara Lara (formerly at CINVESTAV-IPN, Campus Guanajuato, Mexico); a pA plasmid clone containing a 7.3-kb *Eco*RI subfragment of human rRNA genes spanning the 5.8 plus 28S subunits, which was provided by J.E. Sylvester (University of Pennsylvania, PA USA); and pNEBUC GE77+, which is related to the pMF1-c plasmid and was provided by Dr. Michael Feldbrügge (Institute of Microbiology, Heinrich-Heine-Universität, Düsseldorf, Germany) [47].

Table 3 lists the *U. maydis* strains used. The *U. maydis* sexually compatible strains 520 (*a2*, *b2*, *nar1-1*, and *met1-2*) and 521 (*a1* and *b1*) were the parental strains. All of the fungal strains were grown at 30°C in the following culture media: potato dextrose broth (PDB) or agar (PDA) from Difco (Becton Dickinson and Company, Sparks MD, USA); YEPS medium [1% yeast extract (Difco), 2% Bacto peptone (Difco), and 2% sucrose]; complete (CM) or minimal (MM) medium as previously described [48]; and mating medium containing 1% active charcoal [23]. Media were supplemented with either 150 or 200 µg/ml hygromycin (Invitrogen) as specified or with 1 µg/ml carboxin (Cbx; BioWorld, Dublin, OH, USA) when necessary. *Fuz+* mating assays were performed on charcoal nutrient medium as previously described [23], [48].

### Plant infection with *U. maydis*

Plant infection was achieved as previously described [48], [49]. *U. maydis* cells were grown in YEPS medium (1% yeast extract, 1% peptone, and 2% sucrose) at 28°C for 14 h. Mixtures of compatible strains were prepared, and 100 µl of these cell suspensions was injected into five-day-old maize seedlings (MCS-02) [49]. The tumor tissues were harvested at 15–30 days post-inoculation (dpi), processed or frozen, and stored at −70°C until nucleic acid extraction. To recover the progeny, mature teliospores were germinated on solid medium supplemented with hygromycin (150 µg/ml) when necessary [48]. After germination, the sporidia were re-streaked in duplicate on medium supplemented with and without hygromycin (150 µg/ml). Progeny strains were grown on MC supplemented with hygromycin when needed and stored in a solution 15% (v/v) glycerol at −70°C until analysis.

### Nucleic acid manipulation

DNA was prepared from 20 ml of *U. maydis* culture grown O/N. Protoplasts of the harvested cells were prepared as described in [50] using 20 mg/ml of Lysing Enzymes from *Trichoderma harzianum* (L1412; Sigma-Aldrich Co, St. Louis, MO, USA). The protoplasts were collected, and 1 ml of extraction buffer (200 mM Tris-HCl, pH 8.5; 250 mM NaCl; 25 mM EDTA; and 0.5% SDS) was added to the cell pellet and mixed to homogeneity. Phenol-chloroform extraction was conducted twice with 15-min centrifugation intervals to purify the DNA. The aqueous phase was washed once with chloroform and precipitated with 0.6 v/v isopropanol. The DNA was recovered and suspended in 100 µl of sterile distilled water and re-extracted with phenol. The DNA was then suspended in sterile distilled water.

For Southern blot assays, the DNA was quantified prior to and after restriction enzyme digestion with a VersaFluor Fluorometer System (Bio-Rad) using Hoechst 33258 (Fluorescent DNA Quantitation Kit, Bio-Rad) according to the manufacturer's instructions. Equal amounts of DNA were used for each lane in the assays.

To prepare total RNA, *U. maydis* cultures were grown to an O.D._600_ of 0.6. Sporidia were washed with PBS, centrifuged, and suspended in 3 ml of 20 mM sodium acetate buffer (pH 5.5); we then proceeded as previously described [51], [52]. After the final precipitation step, RNA was treated with RNase-free DNase, extracted twice with phenol-chloroform, and precipitated. The RNA was quantified by measuring the UV absorbance at 260 nm and assessed by PCR to detect any contaminating DNA.

Samples of RNA were treated with RNase-free recombinant DNase I (Roche, Mannheim, Germany) to remove contaminating DNA following the manufacturer's recommendations. Reverse transcription was performed using RevertAid H minus Reverse Transcriptase (PureExtreme, Fermentas, USA) and 1 µg DNA-free RNA, according to the manufacturer's instructions. Five microliters of synthesized cDNA was used as a template for PCR amplification with the CGFMD05 (5′ GTCTTTCCGCATTACGTCTACAGG 3′) and FATCR08 (5′ TCGCCTTTGCTGGAGCCTTTACG 3′) primers. Primers were designed based on the *trt1* sequence, and they produced a 1222-bp product after amplifying DNA sequences spanning from gene positions 411 to 1633 nt. After a 5-min 94°C denaturation period, 25 amplification cycles were performed as follows: 70°C for 30 s, 72°C for 1 min 40 s, and 92°C for 50 s. PCR reactions were performed using Platinum *Taq* DNA Polymerase (Invitrogen) according to the manufacturer's instructions.

### *U. maydis* transformation and one-step *trt1* disruption

The transformation procedure was performed essentially as described [50], using protoplasts and polyethylene glycol 3350. The regeneration plates were prepared with MM or CM [48] supplemented with 1 M sorbitol and either 200 µg/ml hygromycin (Sigma-Aldrich) or 1 µg/ml carboxin (BioWorld) as selection agents. The transformed cells arose as hygromycin- or carboxin- resistant colonies after five days of incubation at 28°C. To analyze the senescence phenotype of the hygromycin-resistant transformants, single colonies from the transformation plates were streaked on agar or grown for up to 36 doubling periods in Holliday's MM under hygromycin (200 µg/ml) selection. The cultures were used for DNA extraction, and aliquots were stored in glycerol at −70°C.

For the one-step disruption of the putative *trt1* gene, a ∼4,230-bp linear *Eco*RV fragment was obtained from the pCM1007 plasmid, which contains a disruption cassette. The cassette was an assembly of the bacterial hygromycin phosphotransferase gene (*hph*) expressed under the control of the P_hsp70_ *U. maydis* promoter. This marker gene was linked to two flanking fragments of the *trt1* gene, an 847-bp fragment (from -11 to 836 nt) and a 1,334-bp fragment (from 2,777 to 4,111 nt), as suitable sequences for homologous recombination. The oligonucleotide primer pairs used to amplify these *trt1* DNA fragments were EST2Afor (5′ GAAGAACTGAGATGCAGCC 3′) with EST2Arev (5′ CTGATTCGAGAAGTGACTCG 3′) for the 847-bp DNA fragment and EST2Bfor (5′ CGAGATCATCTTCGGCTTGC 3′) with EST2Brev (5′ TTGACAAGGACTTCTGAGCG 3′) for the 1,334-bp DNA fragment. Multiplex PCR amplification with three oligonucleotide primers (EST2 FDD1, EST2 RDD1, and HyRDD, provided by W.K. Holloman) distinguished between the wild-type and disrupted transformants. The EST2 FDD1 (5′ GGTACTTGCTTTGTGCAGC 3′) and EST2 RDD1 (5′ CTAACGACTCGACTTCAGC 3′) primer pair amplified a 904-bp *trt1* fragment spanning from positions 595 to 1,498 nt. Additionally, the primer combination of HyRDD1 (5′ TCAGGCTCTCGCTGAATTCC 3′) and EST2 FDD1 amplified a fragment of approximately 1,286 bp that spanned part of the *trt1* gene and part of the *hph* gene. A third primer pair of HyRDD1 and EST2FDD2 (5′ TTGCGCAACTTCCTACGAGACT 3′, positioned from −62 nt to −40 of *trt1*) amplified a 1,942-bp DNA fragment that confirmed the *trt1* disruption in the mutants. Two independent clones derived from the *U. maydis* 521 strain (trt1-*1* and trt1-2) were obtained and used for subsequent analysis; one independent clone derived from the 520 strain (520trt1-1) was also used in this study (Table 3).

### Cloning *trt1* under control of the *HSP70* promoter

The forward primer ExTelF00 (5′ ATGCAGCCACCCAAATCCAG 3′) and the reverse primer ExTelRv1 (5′ GCAGCTGGCACGTTGACACA 3′) were used to amplify entire ORF of the *trt1* gene (positions 1 to 4,116 plus 58 bp of the downstream sequence). Thirty PCR cycles were performed with Platinum *Taq* DNA Polymerase (Invitrogen) according to the manufacturer's instructions. In each cycle, the sample was denatured at 94°C for 30 s, annealed at 61°C for 30 s, and extended at 72°C for 5 min. The pTrt1 vector is a pNEBUC GE77+ derivative that contains *trt1* under the control of P_HSP70_ and T_HSP70_. This vector was constructed as detailed below. An amplified 4,171-bp fragment spanning the entire *trt1* ORF was inserted into the *Eco*RV site in pBluescript KS-. The resulting plasmid, pTelMI, was linearized with *Bam*HI and partially digested with *Xho*I to retrieve *trt1*, which was then inserted into the pTrcHis 2A (Invitrogen) plasmid to obtain the pTelMII plasmid. A 2.1-kb *Hind*III fragment from the pUBLEX1 vector carrying the P_HSP70_ promoter and T_HSP70_ terminator sequences separated by a *Bgl*II site was subcloned into the pBluescript KS- vector (Stratagene) to obtain pSVM1. A 4.2-kb *Bam*HI/*Bgl*II (partial digestion) fragment from the pTelMII plasmid carrying the *trt1* sequences was inserted into the *Bgl*II site of pSVM1 in the proper orientation to obtain pTelM70. The *Bam*HI/*Kpn*I fragment encoding Trt1 under the control of the HSP70 promoter and terminator was subcloned into the replicative pNEBUC GE77+ vector to obtain pTrt1.

After transforming *U. maydis* strains with the plasmid, total DNA was isolated from transformant clones, and the oligonucleotide pair CGFMD05 and HSPseqD (5 GCGTCTGTGCGGCGATGAG 3′, located at position 605 to 623 nt of the 786-bp HSP70 promoter) was used to detect the chimeric gene by amplifying a 1813-bp DNA fragment spanning part of the P_HSP70_ promoter and part of the *trt1* ORF gene.

### The TRF analysis in *U. maydis*

This analysis was conducted as previously described by Sanchez-Alonso *et al*. [31]. DNA samples were digested to completion with *Pst*I (Invitrogen). DNA fragment size fractionation was performed via electrophoresis on a 1.0% agarose gel, and the resolved products were transferred to a nylon membrane (Hybond-N, Invitrogen) as described elsewhere [51]. Hybridization was performed at 65°C in Church's buffer at a probe activity of 17 kBq/ml [53]. One of the probes used in this study was UT1-a, which spans 240 bp of the telomeric repeat sequence (TTAGGG)_39_ plus 329 bp from a highly conserved subtelomeric sequence composed of the TR-p + TR-d fragments [31]. UT1-a was digested to completion with *Hinc*II (Invitrogen) to separate the telomere repeat sequence (TTAGGG)_n_ from the adjacent subtelomeric sequences to use as individual probes. The UT4-a probe, which is derived from the subtelomeric *UTASa* (Accession AF030885.1), was also used in the hybridization assays [32]. Probes were labeled using the Random Primers DNA Labeling System (Invitrogen) and used as previously described [32]. Washes were conducted at 25°C and included two washes with 5× SSPE and 0.2% SDS and one wash with 2× SSPE and 0.1% SDS, with gentle shaking for 15 s. When necessary, additional washes with 2× SSPE or 0.4× SSPE plus 0.1% SDS were performed. To reuse the membranes, the probes were removed by gently shaking the membrane in a boiling solution of 0.1% SDS and 0.1× SSPE for 1 min. The solution was then discarded, and this treatment was repeated at least twice (20× SSPE: 3 M NaCl, 0.2 M NaH_2_PO_4_•H_2_O, 0.02 M EDTA, and pH = 7.4).

### The telomeric repeat amplification protocol (TRAP) in *U. maydis*

To measure the telomerase activity we used the TRAP, which has been described previously by Kim *et al*. [54]. To obtain protein extracts from different strains, 1000- ml cultures were grown to a D.O._600_ of 0.6. Cells were harvested by centrifugation, washed once in PBS, and resuspended at approximately 10^9^ cells/ml in lysis buffer containing the following: 10 mM Tris-HCl (pH 7.5), 1 mM MgCl_2_, 10% glycerol, 1 mM EGTA (Sigma-Aldrich, St. Louis, MO, USA), 0.5% Tween 20, and 1 mM dithiothreitol (Amersham Biosciences, Piscataway, NJ, USA). The protease inhibitor cocktail described by Masutomi *et al*. [55] was used, with slight modifications as follows: the inhibitors were used at one-tenth the prescribed concentration, the benzamidine HCl concentration was increased to 160 µg/ml (0.1 mM), the phenanthrorin was replaced with 5 µg/ml chymostatin (Sigma), and 50 U/ml of the RNase inhibitor SUPERase•In (Ambion, Austin, TX, USA) was added. The mixture was frozen in liquid nitrogen, ground to a powder, mixed with 200 µl of cold lysis buffer, and incubated on ice for 30 min. The lysates were centrifuged for 45 min at 25,000×*g* at 4°C, and the supernatant was divided into aliquots, frozen in liquid nitrogen, and stored at −70°C until use. Galls (from 12-day-old plants) and maize leaves or meristems (from 20-day-old plants) were frozen in liquid nitrogen or ground to powder following the same protocol. To determine telomerase activity, the sample protein concentrations were adjusted to 0.1 µg/µl with lysis buffer, and 0.1 µg of the protein extract was poured into a tube containing elongation buffer [56], which was modified with 30 mM Tris-HCl (pH 8.3) and 0.25 mg BSA. The reaction mixture was brought to a final volume of 20 µl and incubated for 30 min at 30°C. To amplify the elongated G-rich strand of telomere repeats, 10 µl of the prior mixture was used for PCR using FastStart Taq DNA polymerase (Roche), according to the manufacturer's instructions. The mixture was subjected to 30 cycles of 95°C for 30 s, 50°C for 30 s, and 72°C for 1 min using a TS primer (5′ AATCCGTCGAGCAGAGTT 3′) plus a CX (5′ CCCTAACCCTAACCCTAACCCTAA 3′) reverse primer. The supplied HEK293 cell line extracts were heat-inactivated at 85°C for 15 min and used as negative controls. Heat-inactivated extracts of *U. maydis* strains or RNase-treated samples were also used as telomerase-negative fungal controls. The Telo*TAGGG* Telomerase PCR ELISA kit (Roche) was used to determine telomerase activity accordingly to manufacturer's instructions. The average absorbance value (A) for each sample was calculated as recommended, A = A_450_ (reading) - A_690_ (blank). To calculate the difference in absorbance (ΔA), we subtracted the mean of the absorbance of the negative controls from the mean sample absorbance. The samples were identified as telomerase positive if the difference in absorbance (ΔA = A_sample_ - A _neg. control_) was higher than 0.2 units. Further details of the manufacturer's protocol can be found in the *T*elo*TAGGG* Telomerase PCR ELISA manual (V8.0 p.15). Statistical analyses were conducted using the Mann-Whitney test, which was designed to compare differences between two independent groups [57]. The GraphPad Prism V.6.0b program (GraphPad Software, La Jolla, CA, USA) was used following the manufacturer's instructions (www.graphpad.com/guides/prism/5/user-guide/prism5help.html?stat_how_to_do_an_unpaired_t_test_w_3.html). Figures 4 and S1D were created with GraphPad Prism V.6.0b using results of the Kruskal-Wallis test (one-way ANOVA data test), which is an extension of the Mann-Whitney U test used to compare two or more samples.

### Exonuclease digestion of *U. maydis* DNA

The assay was performed with *Bal*31 exonuclease (Takara Bio, Shiga, Japan) according to the manufacturer's instructions. Briefly, 1 µg of total DNA was adjusted to 250 µl with the 1× *Bal*31 nuclease buffer provided by the manufacturer. Samples (62 µl) were removed at regular intervals depending on the strain. The reactions were terminated by adding EGTA (Sigma) to a final concentration of 10 mM. The samples were ethanol precipitated, and the DNA was digested with *Pst*I (Invitrogen) and electrophoresed on 1% agarose gels. The samples were then transferred to nylon membranes (GeneScreen Plus, Hybridization Transfer Membrane, Perkin Elmer Inc., Alameda, CA, USA) and hybridized to (TTAGGG)_n_, UT4-a, both, or rRNA gene sequences as a probe.

## Supporting Information

Figure S1 ***Tert1*** **expression in complemented** ***U. maydis*** **strains.** (A) After transformation with pTrt1, the T1 clone (third row) was grown under hygromycin and carboxin selection in Holliday's minimal medium (MM) plates to verify plasmid acquisition. Wild-type 521 (first row), non-transformed W204 (second row), and pNEBUC GE77+ transformed clone from the 521 strain (fourth row) were used to compare the drug resistance profile. Dilutions of strains were performed to obtain 10^5^ to 10^1^ cells in poured drops, and MM was used as a control to avoid false negatives. (B) The DNA sequences in pTrt1, spanning part of the *trt1* ORF and part of the P_HSP70_ promoter, were amplified by PCR to verify plasmid acquisition into the cells. The panel shows the amplification products of the 521 strain (lane 1), plasmid pTrt1 (lane 2), trt1-1 mutant (lane 3), the W204 strain (lane 4) and five of their transformant clones (T1 to T5). (C) The transcriptional activity from the chimeric *tert1* gene was measured by RT-PCR assays in three of the aforementioned transformant clones. Mock RT-PCR assays lacking reverse transcriptase (lane 1) or RNA from the tested 521 strain (lane 2) served as an example of the procedure used to reveal traces of contaminating DNA. Amplified cDNA from *tert1* transcripts from the 521 strain (lane 2), trt1-1 mutant (lane 4), the W204 strain (lane 5) and three of their transformant clones (T1 to T3) are shown. In (B) and (C), the molecular weight marker is on the left. (D) Telomerase activity in the wild-type and pTrt1 complemented strains was determined by TRAP as before (Fig. 4). The graphical representation of *U. maydis* telomerase activity was constructed with GraphPad Prism V.6.0b. The strain names included in the assay are shown at the x-axis. Significant differences were detected between telomerase-complemented and telomerase-negative strains (P<0.05).
(TIF)

Figure S2 **Determining TRF by** ***Bal*** **31 exonuclease analysis in telomerase-negative** ***U. maydis*** **strains.** To confirm TRF sensitivity to *Bal*31 exonuclease in the analyzed strains, we digested total DNA from the selected strains shown in Figure 7. The following strains were used: (A) and (B) the wild-type 521 strain, (C) the disrupted trt1-1 strain, (D) the post-meiotic F1 W204 strain, and (E) the transformant T2. For 521, trt1-1 and T2, 2 U/µg DNA of Bal31 exonuclease was used, and for W204, 6 U/µg DNA of exonuclease was used. The digestion times are indicated above the panels, and the strain name is indicated higher up; the probe used is indicated below the panels. Arrows indicate the size-changes in the hybridization signals in the disrupted and complemented strains. The molecular weight is indicated on the left.
(TIF)

Table S1 **Telomerase activity in** ***U. maydis*** **complemented strains.** The average values of the absorbance in cell extracts were calculated according to the manufacturer's instructions. The values obtained for each sample are presented. Negative controls for HEK293 consisted of heat- (85°C) and RNase-treated samples. Telomerase activity was measured under the same conditions in the positive controls and tested samples. N.D. indicates that the absorbance was not determined. All of the experiments were performed at least three times.
(XLSX)
