# Supplementary figures and images for: The Telomerase Reverse Transcriptase Subunit from the Dimorphic Fungus Ustilago maydis
Source: PLoS One. 2014 Oct 9;9(10):e109981. doi: 10.1371/journal.pone.0109981 (PMC4192592; doi:10.1371/journal.pone.0109981)

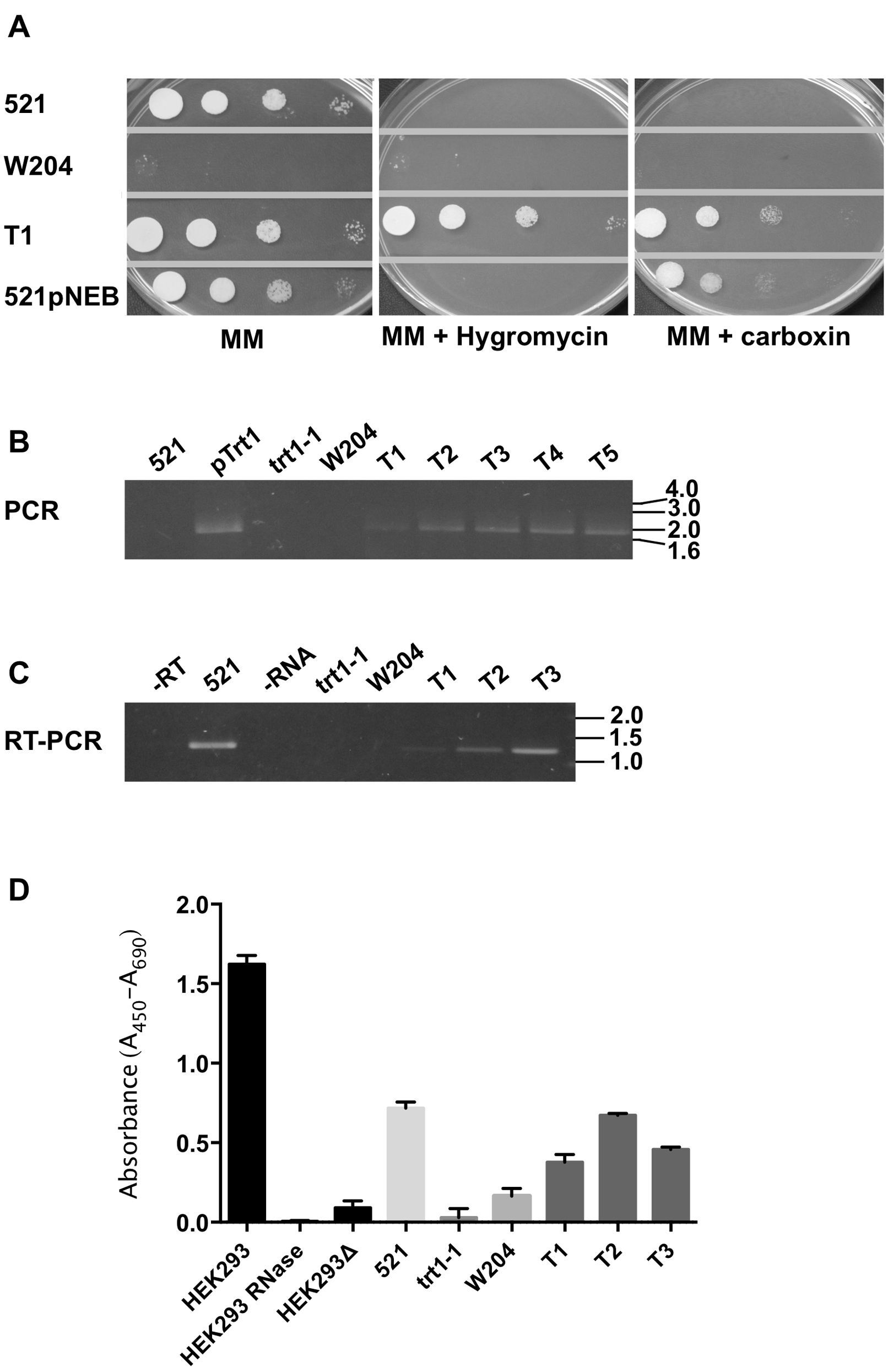

Supplement: Figure S1 — Tert1 expression in complemented U. maydis strains. (A) After transformation with pTrt1, the T1 clone (third row) was grown under hygromycin and carboxin selection in Holliday's minimal medium (MM) plates to verify plasmid acquisition. Wild-type 521 (first row), non-transformed W204 (second row), and pNEBUC GE77+ transformed clone from the 521 strain (fourth row) were used to compare the drug resistance profile. Dilutions of strains were performed to obtain 105 to 101 cells in poured drops, and MM was used as a control to avoid false negatives. (B) The DNA sequences in pTrt1, spanning part of the trt1 ORF and part of the PHSP70 promoter, were amplified by PCR to verify plasmid acquisition into the cells. The panel shows the amplification products of the 521 strain (lane 1), plasmid pTrt1 (lane 2), trt1-1 mutant (lane 3), the W204 strain (lane 4) and five of their transformant clones (T1 to T5). (C) The transcriptional activity from the chimeric tert1 gene was measured by RT-PCR assays in three of the aforementioned transformant clones. Mock RT-PCR assays lacking reverse transcriptase (lane 1) or RNA from the tested 521 strain (lane 2) served as an example of the procedure used to reveal traces of contaminating DNA. Amplified cDNA from tert1 transcripts from the 521 strain (lane 2), trt1-1 mutant (lane 4), the W204 strain (lane 5) and three of their transformant clones (T1 to T3) are shown. In (B) and (C), the molecular weight marker is on the left. (D) Telomerase activity in the wild-type and pTrt1 complemented strains was determined by TRAP as before (Fig. 4). The graphical representation of U. maydis telomerase activity was constructed with GraphPad Prism V.6.0b. The strain names included in the assay are shown at the x-axis. Significant differences were detected between telomerase-complemented and telomerase-negative strains (P<0.05). (TIF) [file pone.0109981.s001.tif]

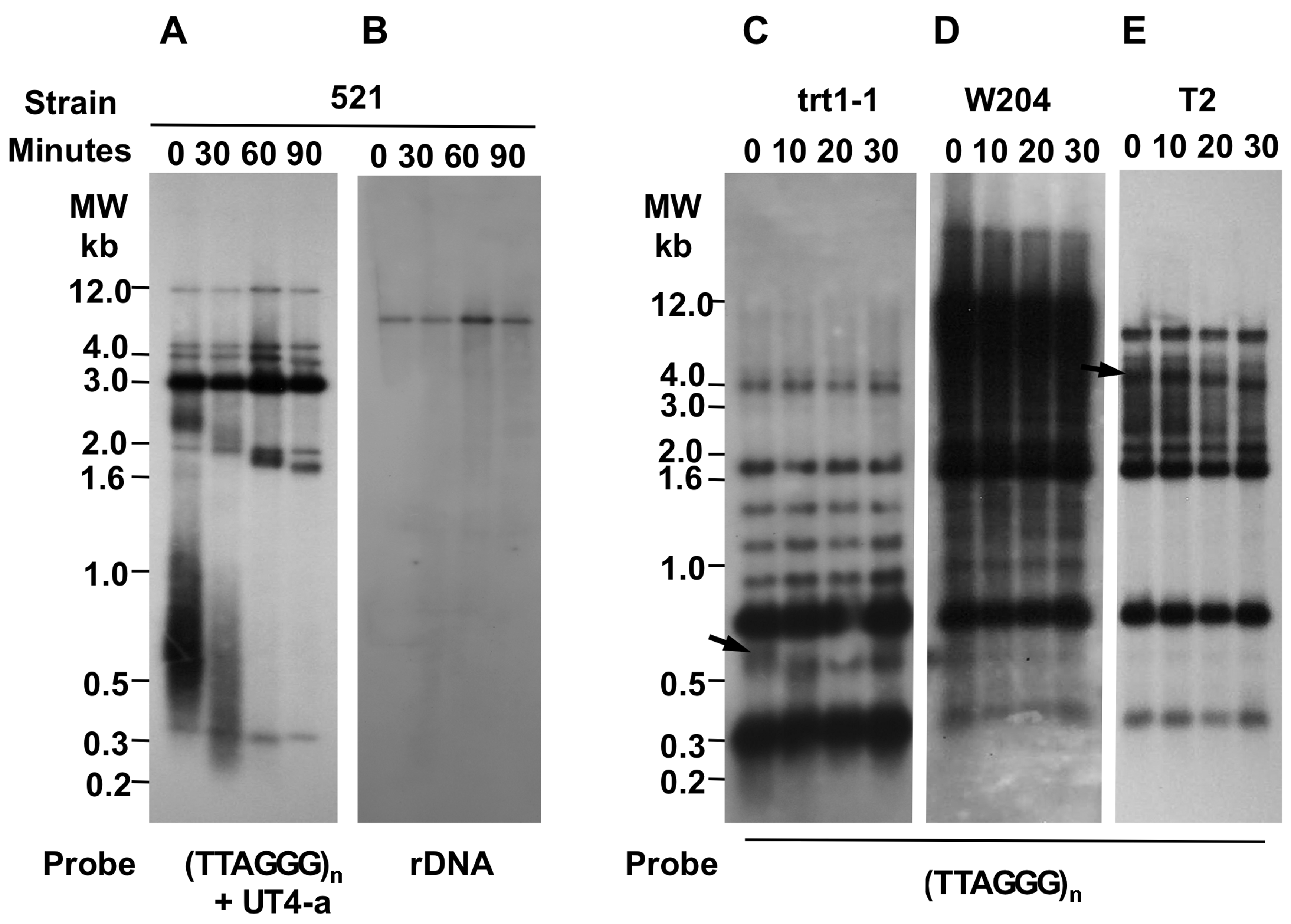

Supplement: Figure S2 — Determining TRF by Bal 31 exonuclease analysis in telomerase-negative U. maydis strains. To confirm TRF sensitivity to Bal31 exonuclease in the analyzed strains, we digested total DNA from the selected strains shown in Figure 7. The following strains were used: (A) and (B) the wild-type 521 strain, (C) the disrupted trt1-1 strain, (D) the post-meiotic F1 W204 strain, and (E) the transformant T2. For 521, trt1-1 and T2, 2 U/µg DNA of Bal31 exonuclease was used, and for W204, 6 U/µg DNA of exonuclease was used. The digestion times are indicated above the panels, and the strain name is indicated higher up; the probe used is indicated below the panels. Arrows indicate the size-changes in the hybridization signals in the disrupted and complemented strains. The molecular weight is indicated on the left. (TIF) [file pone.0109981.s002.tif]
